# Examination of molecular space and feasible structures of bioactive components of humic substances by FTICR MS data mining in ChEMBL database

**DOI:** 10.1038/s41598-019-48000-y

**Published:** 2019-08-19

**Authors:** Alexey A. Orlov, Alexander Zherebker, Anastasia A. Eletskaya, Viktor S. Chernikov, Liubov I. Kozlovskaya, Yury V. Zhernov, Yury Kostyukevich, Vladimir A. Palyulin, Eugene N. Nikolaev, Dmitry I. Osolodkin, Irina V. Perminova

**Affiliations:** 1 FSBSI “Chumakov FSC R&D IBP RAS”, Moscow, 108819 Russia; 2 Skolkovo Institute of Science and Technology, Moscow, 143026 Russia; 3 Department of Chemistry, Lomonosov Moscow State University, Moscow, 119991 Russia; 4 Department of Fundamental Medicine, Lomonosov Moscow State University, Moscow, 119991 Russia; 5 Sechenov First Moscow State Medical University, Moscow, 119991 Russia; 6 State Research Center “Institute of Immunology” of the Federal Medical-Biological Agency of Russia, Moscow, 115478 Russia

**Keywords:** Cheminformatics, Screening

## Abstract

Humic substances (HS) are complex natural mixtures comprising a large variety of compounds produced during decomposition of decaying biomass. The molecular composition of HS is extremely diverse as it was demonstrated with the use of high resolution mass spectrometry. The building blocks of HS are mostly represented by plant-derived biomolecules (lignins, lipids, tannins, carbohydrates, etc.). As a result, HS show a wide spectrum of biological activity. Despite that, HS remain a ‘biological activity black-box’ due to unknown structures of constituents responsible for the interaction with molecular targets. In this study, we investigated the antiviral activity of eight HS fractions isolated from peat and coal, as well as of two synthetic humic-like materials. We determined molecular compositions of the corresponding samples using ultra-high resolution Fourier-transform ion cyclotron resonance mass-spectrometry (FTICR MS). Inhibitory activity of HS was studied with respect to reproduction of tick-borne encephalitis virus (TBEV), which is a representative of *Flavivirus* genus, and to a panel of enteroviruses (EVs). The samples of natural HS inhibited TBEV reproduction already at a concentration of 1 µg/mL, but they did not inhibit reproduction of EVs. We found that the total relative intensity of FTICR MS formulae within elemental composition range commonly attributed to flavonoid-like structures is correlating with the activity of the samples. In order to surmise on possible active structural components of HS, we mined formulae within FTICR MS assignments in the ChEMBL database. Out of 6502 formulae within FTICR MS assignments, 3852 were found in ChEMBL. There were more than 71 thousand compounds related to these formulae in ChEMBL. To support chemical relevance of these compounds to natural HS we applied the previously developed approach of selective isotopic exchange coupled to FTICR MS to obtain structural information on the individual components of HS. This enabled to propose compounds from ChEMBL, which corroborated the labeling data. The obtained results provide the first insight onto the possible structures, which comprise antiviral components of HS and, respectively, can be used for further disclosure of antiviral activity mechanism of HS.

## Introduction

Antiviral therapy presents a great challenge for drug discovery because the mutation rate of viruses is high and new pathogenic strains and species emerge quickly^1^. Many viral genera including important human pathogens are devoid of small molecule antivirals. For example, *Flavivirus* genus members, such as dengue virus (DENV), tick-borne encephalitis virus (TBEV), Zika virus (ZIKV), etc., are not manageable with drugs^2^. TBEV is the leading cause of arbovirus infections in Europe and Russia^3^. Several small molecule classes were suggested as the starting compounds for the development of TBEV reproduction inhibitors^4–13^. However, none of them were developed as drug candidates and there is still a need for new anti-TBEV compounds.

Natural products are actively used in drug design due to their pronounced physiological activity and accessibility^14^. They are thought to possess structures fitted for interactions with proteins, lipids, etc., thus being promising starting points for drug design^15^. Humic substances (HS) represent an example of complex natural mixtures containing diverse organic acids produced by oxidative degradation of biomacromolecules such as terpenoids, lignins, polysaccharides, peptides, tannins, etc.^16–18^. HS are characterized by broad spectrum of biological activity, including antibacterial, antiviral, and anti-inflammatory properties^19^. Molecular understanding of HS was achieved by Fourier transform ion cyclotron resonance mass-spectrometry (FTICR MS) with soft electrospray ionization (ESI)^20,21^ Due to its high resolution, this method detects thousands of molecules in complex mixtures without preliminary fractionation^22,23^. However, FTICR MS analysis is limited by its inability to distinguish structural isomers^24^. Only a general knowledge of the structures comprising HS can be obtained from the elemental compositions of the ionizable molecules without feasible tandem mass-spectrometric identifications. A conventional approach to the analysis of HS is to assign structural motifs to molecular compositions with respect to their major precursors with similar atomic ratios^25^ or double bond equivalent^26^. Deeper structural study using fragmentation approaches is hampered by a lack of suitable preliminary separation techniques and extreme diversity of molecular compositions, which results in the increased amount of molecular formulae assignments after HS fractionation rather than in narrowing of complexity^27^. This leaves the question open with regard to individual HS components responsible for the bioactivity of these complex mixtures.

At the same time, information on structures and properties of compounds isolated from natural complex mixtures is accumulated in large public databases, such as ChEMBL^28^, PubChem BioAssay^29^, and others (e.g. refs.^30,31^), allowing the researchers to repurpose compounds, build pharmacological profiles, QSAR models, etc. Data mining of mass-spectrometric results in chemical databases enables to avoid re-identification of well-known compounds, which is usually referred to a dereplication strategy^32^. Application of such approaches is particularly important for structural studies of biologically active metabolites, which are time-consuming, require high amounts of the parent materials and often lead to the unsatisfactory results. Recently, possible structures comprising oxidized lignin and humic samples were suggested by *in silico* search of molecular formulae revealed by high-resolution mass spectrometry in chemical databases^33,34^. Also, a combination of FTICR MS data mining in PubChem with total statistics of neutral mass losses during fragmentation enabled to suggest a lower estimate for possible isomeric complexity^35^.

The objective of this study was to develop a chemoinformatic approach for the analysis of chemical space of bioactive HS components and exploration of possible structural motifs *via* a search for compounds matching FTICR MS-assigned molecular formulae in ChEMBL database. ChEMBL was chosen as one of the largest and carefully curated databases on chemical structures and biological activities. We also assessed the antiviral activity of ten HS samples from different sources against tick-borne encephalitis virus (TBEV, genus *Flavivirus*) and *Enterovirus* (EV) genus representatives and retrieved ChEMBL compounds with comparable bioactivity profiles. For supporting chemical relevance of the compounds found in ChEMBL to natural HS, we applied the previously developed approach of selective isotopic exchanged coupled to FTICR MS for obtaining structural information on the individual components of HS^36^. The obtained results provide first insight on the possible structures, which comprise antiviral components of HS and, respectively, can be used for further dissection of HS antiviral activity mechanism.

## Results

### Antiviral activity of HS samples

Antiviral activity of the ten HS samples used in this study was assessed by plaque reduction test in PEK cells for TBEV and by cytopathic effect inhibition test in RD cells for a panel of enteroviruses. All HS samples used in this study, except for the two synthetic ones, showed antiviral activity in the EC_50_ range of 0.1–1 μg/mL with pre-incubation of the virus with the samples (TBEV EC_50_pre_, Table 1). However, no activity was observed when the virus and the sample were added to the cells simultaneously (TBEV EC_50_sim_, Table 1). Synthetic samples did not show a detectable activity in either of the experiments. Neither samples show any inhibition of cytopathic effect caused by enteroviruses (EV EC_50_pre_, Table 1).

**Table 1:** Antiviral activity and cytotoxicity of the HS samples used in this study.

Sample	CC_50_				TBEV EC_50_pre_	TBEV EC_50_sim_	EV EC_50_pre_
	PEK		RD				
	24 h	7 d	24 h	7 d			
CHA-GL	>10	>10	10.5	1.8	0.30 ± 0.19	>10	>20
CHA-Pow	>10	>10	7.4	1.8	0.26 ± 0.14	>10	>20
CHA-SH4	>10	>10	14.7	3.7	0.74 ± 0.15	>10	>20
CHM-GL	>10	>10	14.7	1.8	0.514 ± 0.025	>10	>20
CHM-Irk	>10	>10	14.7	1.8	0.14 ± 0.08	>10	>20
CHM-Pow	>10	>10	>20.8	>20.8	0.808 ± 0.016	>10	>20
HQ-FA	>10	>10	ND^[a]^	>10	>10	>10	>20
MHQ-FA	>10	>10	>20.8	>20.8	>10	>10	ND
PHA-T7	>10	>10	14.7	3.7	0.9 ± 0.1	>10	>20
PHA-TTL	>10	>10	14.7	7.4	0.7 ± 0.3	>10	>20
control^[b]^	>120	>120	ND	ND	0.39 ± 0.11	ND	ND

^[a]^ND — not determined.

^[b]^3-amino-7,7-dimethyl-2-(4methylbenzoyl)-5H,6H,7H,8H-selenopheno[2,3-*b*]quinolin-5-one^5^.

All values are in μg/mL.

### Analysis of HS samples’ molecular composition

To elucidate a possible relationship between molecular composition and antiviral activity of HS, we analyzed distribution of formulae among the HS samples used in this study (Table 2). It should be noted that the samples of natural origin used in this study included nitrogen-containing compounds. However, according to the elemental analysis, the nitrogen content did not exceed 3% (wt) (Supplementary Table S1). We observed no correlation between EC_50_ values and the nitrogen content. This motivated us to exclude CHON molecular compositions from the consideration, which simplified the further data analysis. We identified 2380 unique molecular formulae, which we defined as formulae present only in a single sample. There were 4122 shared formulae (present in at least two samples), giving in total 6502 different formulae present in the HS samples used in our study. All samples contained 100 common formulae. In addition, 13 formulae were present in the eight natural HS samples studied, but not in the two synthetic ones. It should be noted that without a use of additional information these formulae may be assigned to thousands of structures^24^. Comparison with the known molecules characterized by activity profiles enables to suggest a list of putative scaffolds.

**Table 2:** Formula distribution for the HS samples used in this study.

Sample	MS CHO formulae (% of all assignments)	Unique Formulae^[a]^	in ChEMBL^[b]^
CHA-GL	989 (45)	206	151
CHA-POW	2653 (69)	459	307
CHA-SH4	1361 (52)	102	3
CHM-GL	1543 (63)	36	0
CHM-Irk	2597 (77)	124	39
CHM-POW	2748 (81)	39	8
HQ-FA	885 (100)	29	4
MHQ-FA	745 (100)	2	0
PHA-T7	4488 (92)	1332	676
PHA-TTL	2397 (84)	51	10
Total	20,406 (6502 different)	2380	1198

^[a]^Formulae present only in a particular sample.

^[b]^Unique formulae for a sample that were found in ChEMBL.

**Figure 1:**
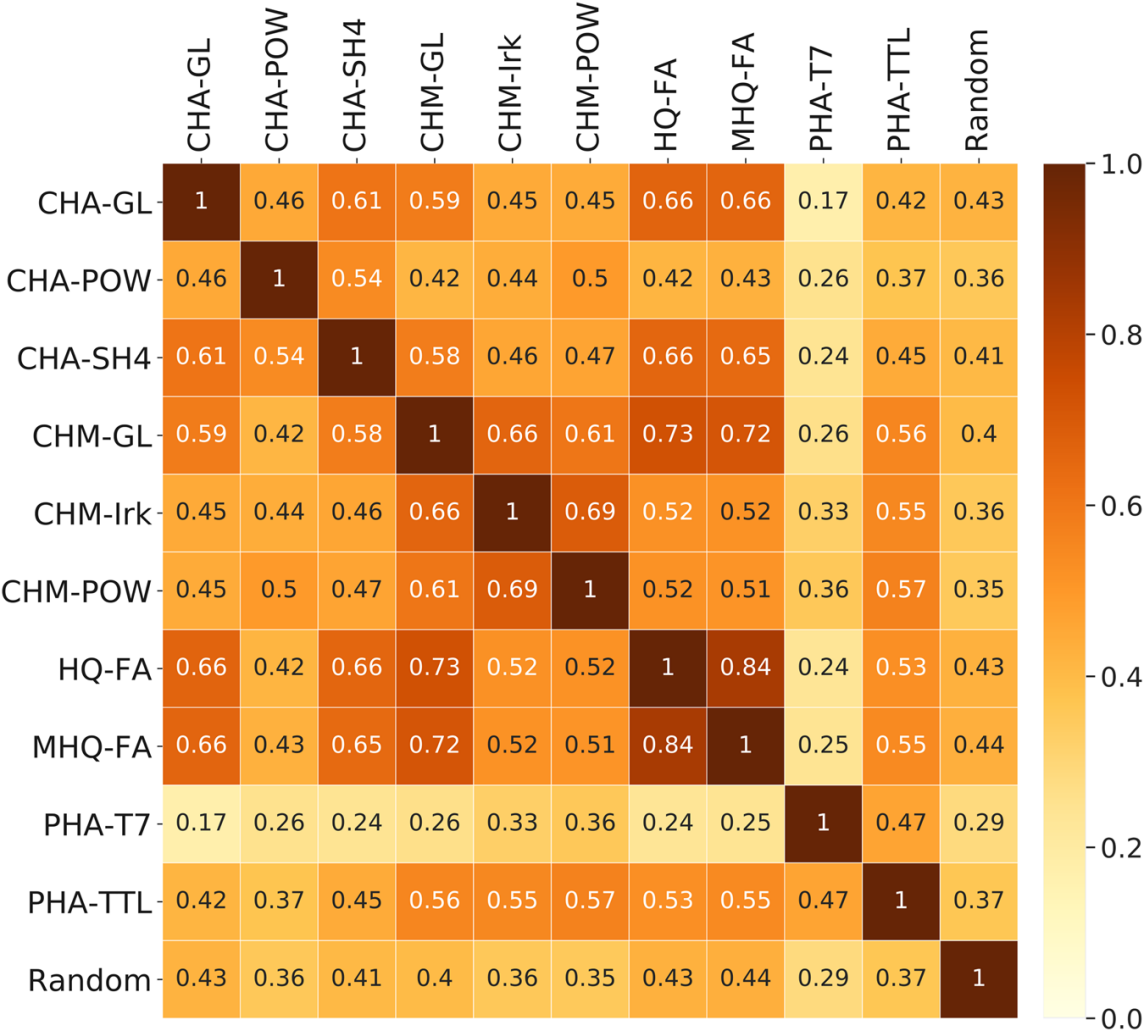
Similarity heatmap for the HS samples used in this study. The Boolean fingerprints were constructed for all samples by setting 1 if the formula was detected in the sample by FTICR MS, and by setting 0 if the formula was not found in the FTICR MS assignments. Coloring and values are Tanimoto indices between fingerprints. The values of similarity with Random fingerprints are means of Tanimoto similarity between the fingerprint in question and 1000 boolean fingerprints containing 2347 randomly positioned ‘1’ values.

Tanimoto similarity heatmap (Fig. 1) was plotted for the HS samples using the Boolean compositional fingerprints. All the samples showed moderate to low similarity to each other. The least active synthetic HS samples (MHQ-FA and HQ-FA) were, on one hand, very similar to each other (the most similar pair of the samples in the set), but on the other hand, they were rather similar to the active samples of coal HS: CHA-GL, CHM-GL, and CHA-SH4. The samples of coal humic acids (CHA) used in our study were very different from their hymatomelanic fractions (CHM), but the samples inside these groups were more similar to each other. The fingerprint with randomly distributed 1 and 0 (‘Random’) values showed uniform similarity of the natural HS samples. The PHA-T7 sample strongly differed from all other samples and had the highest similarity with the Random fingerprint. This sample had the highest internal chemical diversity (Table 2): it contained a factor of 1.6 more formulae than the second most chemically diverse sample, CHM-Pow. It also had much more unique formulae as compared to a combined value for all other samples. This is likely due to the significant contribution of carbohydrates typical for high-moor peat^37^.

**Figure 2:**
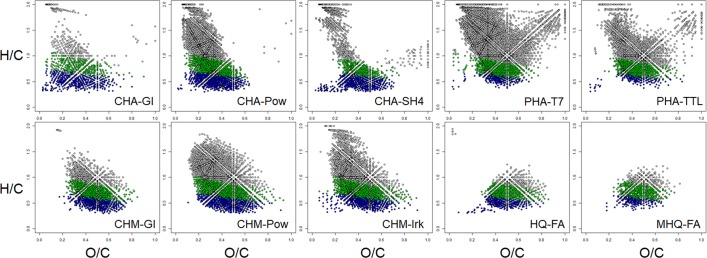
Van Krevelen diagrams of HS samples used in this study. Compounds were designated according to aromaticity index (AI) proposed by Koch *et al*.^26^: condensed with AI ≥ 0.67 (blue), aromatic with AI > 0.5 (green), unsaturated and saturated AI ≤ 0.5 (grey).

For better visualization of HS molecular ensembles, FTICR MS data were plotted on van Krevelen diagrams, representing H/C vs. O/C atomic ratios for all determined formulae (Fig. 2). All coal samples were characterized by the high abundance of low-oxidized aromatic compounds with O/C < 0.5 and H/C < 1. These components may be attributed to condensed tannins or flavonoids^38^. At the same time, fractions of hymatomelanic acids and synthetic HS-like compounds also possessed abundant aromatic species with O/C > 0.5. Except for the CHA-GL and CHM-GL, all the natural HS samples were characterized by a highly populated region with H/C > 1 and O/C < 0.5, which can be related to lignin-like compounds^39^. The peculiarity of peat HS was a presence of saturated oxidized compounds, attributed to carbohydrates. All HA samples were characterized by the appearance of highly saturated low oxidized components (H/C > 1.5, O/C < 0.2), which likely correspond to residual lipids and fatty acids^40^. Visual inspection of van Krevelen diagrams also showed that synthetic HS-compounds were fully depleted with non-aromatic constituents and their molecular ensemble was shifted toward oxygenated compounds.

### Enumeration of HS-like formulae space

**Figure 3:**
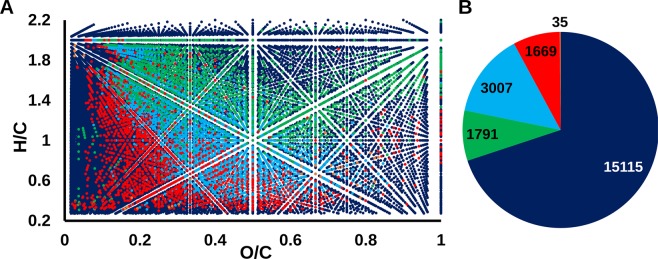
Characterization of the HS samples used in this study and HS-like formulae space. (**A**) Van Krevelen diagram of the formulae identified by FTICR MS in the HS samples used in this study projected onto the HS-like space; (**B**) Assignment of the formulae to the HS origin. Color scheme: all possible C_x_H_y_O_z_ formulae (dark-blue), formulae common for peat and coal (light blue), unique for coal (red), unique for peat (green), unique for the synthetic HS (orange).

Molecular components determined by FTICR MS in the HS samples constitute only a fraction of the total molecular space of humic substances. To approach the probabilistic pool of humic molecular formulae we generated all possible C_x_H_y_O_z_ formulae in the range of 200–800 Da. It yielded 231,546 formulae. Application of atomic constraints (0.27 ≤ H/C ≤ 2.2, 0 < O/C ≤ 1) reported for FTICR MS of HS^25^ has reduced this number down to 22,618 HS-like formulae. For examining existence of molecular graphs, which would correspond to the generated formulae, we applied the Senior’s rules^41–43^. One thousand one of the generated formulae did not satisfy the Senior’s rules filter. As a result, 21,617 formulae were used for the further analysis. The projection of these formulae onto van Krevelen diagram densely covered the whole field (Fig. 3A). All formulae determined for HS samples used in this study were scattered all over the regions of this generated formula space (Fig. 3A) and comprised about 30% of the total HS-like formulae (Fig. 3B, Table 2).

### ChEMBL data mining

**Figure 4:**
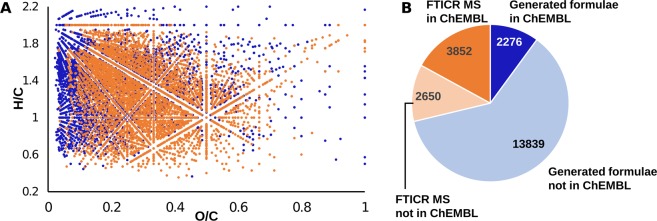
The results of ChEMBL data mining for the generated HS-like molecular formulae and the FTICR MS derived formula assignments for the HS samples. (**A**) Van Krevelen diagram for ChEMBL compounds corresponding to generated HS-like formulae (blue) and FTICR MS derived formula assignments for the HS samples (orange). (**B**) Distribution of the generated and FTICR MS formulae found and not found in ChEMBL. Generated HS-like formulae and FTICR MS derived formula assignments for the HS samples found in ChEMBL are colored blue and orange, respectively. Generated HS-like formulae and FTICR MS derived formula assignments for the HS samples, which were not found in ChEMBL, are colored pale blue and pale orange, respectively.

The ChEMBL data were mined to find the compounds that may be responsible for the anti-TBEV activity of the HS samples. Out of 21,617 generated HS-like formulae, 6128 were found in ChEMBL. These formulae appeared in the central part of the van Krevelen diagram (shown with orange dots in Fig. 4A). About 11% of the generated HS-like formulae were present in the samples, but not found in ChEMBL (Fig. 4B). About 71 K structures and 787 K bioactivity data points were available for 6128 formulae found in ChEMBL. Antiviral activity data points (21,559 entries) linked to 7958 structures were extracted from thoroughly curated subset of antiviral activity data (ViralChEMBL)^44^ to reveal that they were tested against viruses belonging to 25 distinct families. Among the most studied viruses were HIV-1, HCV, and Influenza virus A, from *Retroviridae*, *Flaviviridae*, and *Orthomyxoviridae* families, respectively (Supplementary Fig. S1).

The compounds corresponding to FTICR MS assignments for the HS samples are mostly represented by the typical natural compounds, such as flavonoids, coumarins, and diverse analogues of fungi and plants metabolites. Scaffold analysis (Table 3, Supplementary File S2) shows a substantial enrichment of flavonoid scaffold in the structures related to FTICR MS assignments for the HS samples in ChEMBL and ViralChEMBL subsets, outperforming the benzene scaffold in ViralChEMBL. The compounds related to the generated HS-like formulae, which were not observed in the HS samples by FTICR MS, were represented mostly by cyclic and polycyclic aliphatic scaffolds resembling steroids and betulin. In line with the differences in the most abundant scaffolds we also observed differences in the most abundant functional groups (Supplementary File S2).

**Table 3:** Murcko Scaffold distributions of structures related to formulae present in the HS samples and generated HS-like formulae absent in the HS samples in ChEMBL and ViralChEMBL (% of compounds bearing the scaffold).

Formulae present in the HS samples			Generated HS-like formulae absent in the HS samples		
Murcko Scaffold	No. of compounds	%	Murcko Scaffold	No. of compounds	%
ChEMBL					
	1,903	3.14		802	7.17
	1,180	1.94		162	1.45
	759	1.25		95	0.85
	493	0.81		88	0.79
	380	0.63		83	0.74
ViralChEMBL					
	224	3.27		57	5.83
	191	2.79		20	2.05
	92	1.34		19	1.94
	88	1.28		17	1.74
	76	1.11		14	1.43

There were no data available about activity of the HS-like ChEMBL compounds against TBEV. Since *Flavivirus* genus members are characterized by very similar structure and replication machinery, the activity data for all flaviviruses were extracted. The largest amount of antiviral activity data was available for the dengue virus (DENV). Given that the HS samples were not active against the panel of enteroviruses tested in this study, the structures with these activity features (activity against flaviviruses, inactivity against enteroviruses) were initially retrieved as the most similar to our HS samples by antiviral profile. These compounds were mycophenolic acid and emodin. Mycophenolic acid has isolated double bonds, presence of which in HS is highly unlikely due to their chemical lability. Emodin has never been tested against EV in cell-based assays, according to ChEMBL data, but it did not show activity against Human rhinovirus B protease^45^. We could also find the structures of several inhibitors of flavivirus reproduction or enzymes, which were not tested against enteroviruses. They are listed in Supplementary File S3.

It was previously shown that fractions of single HS samples possess different antiviral activity, which is related to their molecular compositions^18^. Two samples used in our study are subfractions of two others: CHM-POW and CHM-GL were isolated from CHA-POW and CHA-GL samples by exhaustive ethanol extraction in Soxhlet apparatus. For deeper comparison of CHA and CHM molecular ensembles, we performed self-partitioning of the van Krevelen diagrams into 20 cells followed by calculation of intensity-weighted contribution of each cell^46^. Spearman correlation of the cell occupation and the obtained EC_50_ values revealed significant negative correlation for the cells attributed to the polyphenolic structures and to the flavonoid-like ones, in particular (Supplementary Fig. S2)^25^. We believe that increasing intensity of these formulae can reflect an increase in activity of CHA-GL and CHA-POW samples as compared to CHM-GL and CHM-Pow, respectively.

To get deeper insight on possible structures of the compounds populating these regions of van Krevelen diagram, we applied selective isotopic exchange of CHM-Pow, which was chosen among other HS samples because of easy handling. Exchange series were determined for 25 molecular formulae present in CHM-Pow corresponding to the correlated cell. The results are presented in Supplementary Table S2. In all cases we observed at least 1 non-exchangeable oxygen atom, which indicates the presence of ether, alcohol, or ester groups. These results corroborated the amounts of labile hydrogens, which were lower than the number of oxygen atoms. In case of C_22_H_22_O_5_ and C_21_H_22_O_5_ formulae, the number of oxygen atoms and labile hydrogens was equal to five. Taking into account the presence of one non-exchangeable oxygen atom, which must be bound to a hydrogen, this indicates the presence of a single alcohol group in their structures.

Further application of H/D exchange of skeletal protons in DCl revealed aromatic nature of determined compounds with the number of aromatic hydrogens from 2 to 5. After filtration of ChEMBL compounds with regard to the results of isotope exchange FTICR MS for these 25 formulae, 14 structures related to 3 formulae were found (Supplementary File S4).

## Discussion

### Antiviral activity

Antiviral activity of the HS samples used in this study was observed only against TBEV, while none of the samples inhibited enterovirus reproduction in the concentration range up to 20 μg/mL. This is in line with the literature data on polyanionic compounds, which are active against all enveloped viruses, and do not inhibit nonenveloped viruses^47^. This selectivity allowed us to hypothesize on a specific mechanism of anti-TBEV activity of HS samples, presumably, via the inhibition of virus entry into the cell. Due to the anionic nature of HS, this process could be similar to inhibition of flavivirus entry by anionic carbohydrates^48^ mimicking glycosaminoglycans that serve as low-affinity receptors of flaviviruses^49–51^. The common explanation of this phenomenon is that all polyanionic compounds readily interact with positively charged proteins of viral envelope, preventing fusion of virus with the cell. This mode of action was also experimentally supported in our case, when we used an alternative experimental design and added TBEV to the cells simultaneously with HS. In this case, none of the tested HS samples inhibited virus replication even at the highest concentration tested, of 10 μg/mL. This supports the key importance of the virus entry process for the manifestation of HS antiviral activity similarly to the case for all polyanionic compounds.

The highest antiviral activity (EC_50_ 0.14 μg/mL) was observed for hymatomelanic acid isolated from Irkutsk coal, CHM-Irk (Table 1). At the same time, two other coal hymatomelanic acids, CHM-Pow and CHM-GL, had the lower activity (EC_50_ values of 0.8 and 0.5 μg/mL, respectively) as compared to the parent coal humic acids, CHA-Pow and CHA-GL (EC_50_ values of 0.3 and 0.26 μg/mL, respectively). The numeric values of EC_50_ in this study from 0.1 to 0.9 μg/mL are an order of magnitude lower than the corresponding values for the fractions of peloid HS obtained in our previous study of HIV-1 on PMBC cells: from 0.98 μg/mL (HMA) up to 6.7 μg/mL (FA)^18^. In case of the peloid HS tested in our previous study, HMA outcompeted HA fractions in antiviral activity, whereas in this study we observed higher activity of HA versus HMA fractions for the two coal samples. It should be also noted that the peat HA had the lower activity in this study as compared to the coal HA. None of the HS samples showed cytotoxicity for the PEK cell line up to 10 μg/mL; at the same time, they were pronouncedly toxic for the RD cells (Table 1) at concentrations >10 μg/mL after 24 h exposure, and already at 1.8 μg/mL after 7 d exposure. In general, the obtained data show that the antiviral activity of the HS samples depends both on their origin and fractional composition.

Interaction with positively charged proteins of viral envelope is likely one of the several mechanisms of HS biological activity. The visual inspection of van Krevelen diagrams (Fig. 3A) shows an overlap mostly for the low oxidized chemical species in all active samples. Moreover, the formulae unique for coal HS samples are less oxidized compared to peat and synthetic HS, but these are coal HS samples that possess the highest antiviral activity. Therefore, we would rather explain antiviral activity of HS by a combination of hydrophobic and anionic interactions including HS-protein, HS-membrane, HS-RNA interactions, as it was suggested in our previous work^18^.

### Search for HS relevant structures

**Figure 5:**
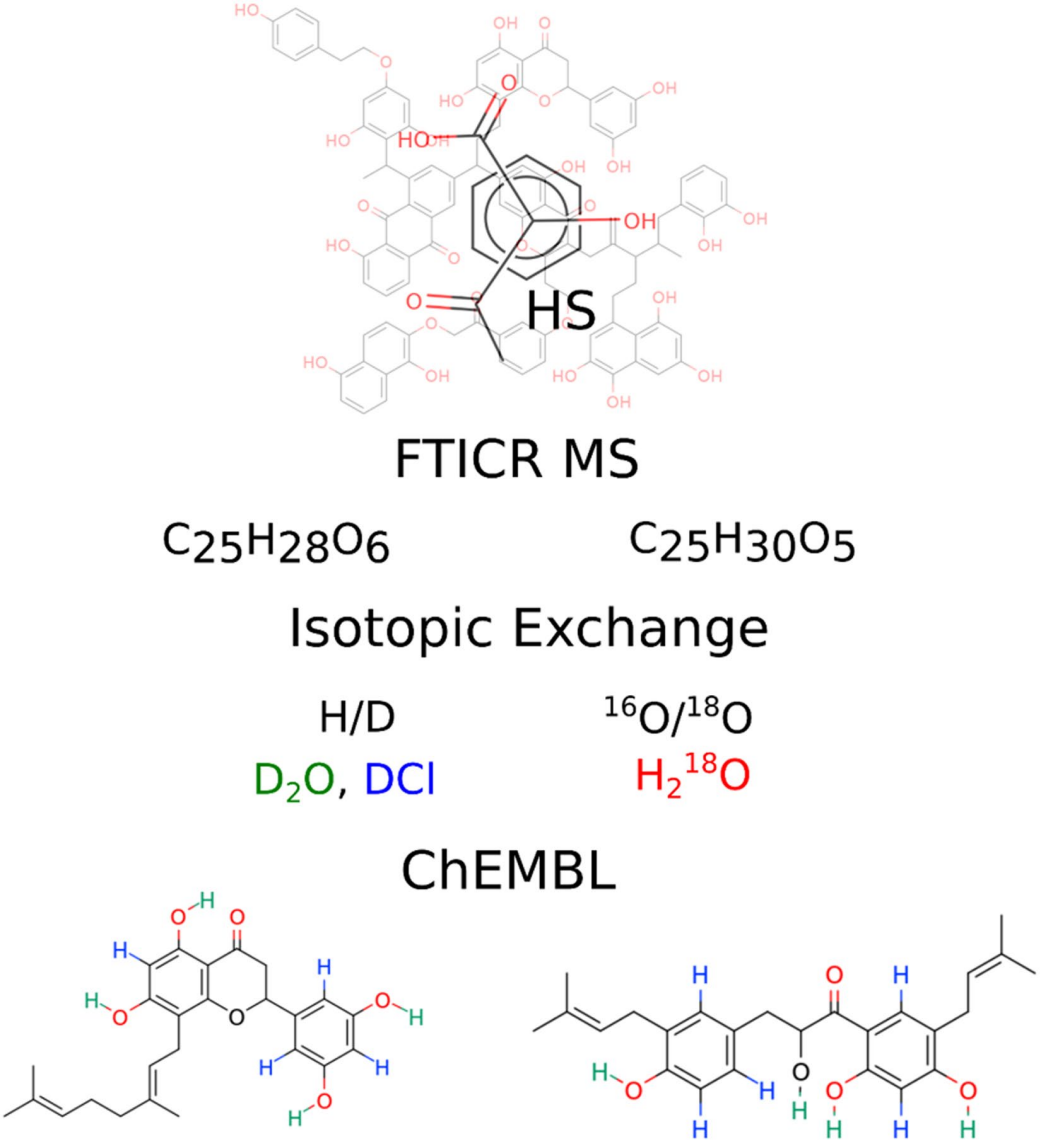
Scheme for the exploration of possible HS components using ChEMBL and FTICR MS isotopic exchange data.

Data mining of HS elemental compositions as well as of the generated HS-like formulae in ChEMBL revealed the dominance of typical humic-like moieties (flavonoids) over aromatic and aliphatic structures. It should be noted that formulae, which were absent in ChEMBL may also drive the humic samples antiviral activity. Nevertheless, the role of flavonoids is highlighted by a significant negative correlation of the corresponding intensity-weighted cells populations in van Krevelen diagram with EC_50_ values for pairs of coal humic acids and their fractions – hymatomelanic acids. At the same time the identified molecular formulae may correspond to a number of isomers^24^. Additional structural information is needed for matching FTICR MS data to ChEMBL. To examine chemical relevance of ChEMBL compounds to natural HS we applied selective isotopic exchange, which enabled identification of the structural features of the individual components of HS. The obtained results were used for filtration of ChEMBL compounds, which match exchange reactions. At the first step we excluded all compounds with the number of labile protons lower than determined. Although all labile protons in HS undergo immediate exchange upon dissolution in D_2_O^52^, we could not exclude back-exchange during ionization^53^, and the actual number of acidic groups (COOH and OH) may be higher as compared to the FTICR MS results. Secondly, we selected all structures, which matched ^18^O/^16^O exchange results. In addition, we filtered out structures, which did not match exchange of aromatic protons in DCl. As a result, we extracted 49 compounds from ChEMBL which possessed partial structural similarity to the HS bioactive components as it is schematically shown in Fig. 5. It should be noted that isomeric complexity of HS components prevents from the suggesting exact matches between them and the found structures. Application of an additional selective modification, such as deuteromethylation^54^, would increase the reliability of isomeric filtration and may facilitate the structure determination for individual HS components. This will increase the chances for deeper understanding of molecular components contribution to HS sample properties.

## Conclusions

In this work, we applied a chemoinformatics approach to make an educated guess about the structural features of compounds that might underlie the antiviral activity of HS. For this purpose, we used the data from publicly available database ChEMBL analyzing HS possible components chemical space and revealing the classes of compounds that might be related to antiviral activity of the HS samples. We have shown that structural features of compounds extracted from ChEMBL did not contradict the data on the selective isotopic exchange reactions, which were applied to one of the HS samples. The further accumulation of data on antiviral activity of HS samples coupled with their fractionation followed by mining of their FTICR MS assigned formulae with isotopoic exchange data constraints in large bioactivity databases can be beneficial for the surmising on the active components of HS.

## Methods

### Chemistry

Humic samples used in this study were acquired from the samples base of the Laboratory of natural humic systems of the Lomonosov MSU (Moscow, Russia) assembled by Dr. I. V. Perminova and coworkers^55^. They were isolated by alkaline extraction according to the International Humic Substances Society (IHSS) protocol^56^. The samples of coal humic acids (CHA) were isolated from three potassium humates commercially produced from leonardite (Powhumus, Germany; CHA-Pow) and lignite (Sakhalin Humate, Russia, and Gumat-80, Russia; CHA-SH4, CHA-Irk), and one sample was extracted from the lignite deposite (Buryatia, Russia; CHA-GL). The samples of peat humic acids (PHA) were extracted from low- and high-moor peat (Tver, Russia), and designated as PHA-TTL and PHA-T7, respectively. The samples of coal hymatomelanic acids (CHM) were isolated by exhaustive ethanol extraction in Soxhlet apparatus from CHA-Pow, CHA-Irk, and CHA-GL as it is described elsewhere^55^. The obtained CHM were designated as CHM-Pow, CHM-Irk, and CHM-GL, respectively. The synthetic humic-like samples (MHQ-FA and HQ-FA) were obtained by oxidative condensation of hydroquinone and 3-(3-methoxy-phenyl)-3-oxopropanoic acid followed by solid phase extraction as described previously^57^. For the antiviral experiments, we used fulvic acid-like fractions (FA) of the synthetic samples because they included most of the aromatic compounds present in the HA-like fractions according to our previous FTICR MS study^57^. In addition, the FA-like samples contained highly oxidized polycarboxylic compounds with O/C > 0.5. Thus, MHQ-FA and HQ-FA possess richer molecular compositions compared to HA-like fractions.

All solid HS samples used in this study were weighted and wetted with 3 M KOH prior to dissolution in water up to a concentration of 1 g/L. High-purity distilled water was prepared using Millipore Simplicity 185 system.

H/D exchange of skeletal protons and ^18^O/^16^O exchange were performed as described in our previous works^20,57^. In brief: mixtures of 5 mg of CHM-Pow with 300 μl of 16% DCl in D_2_O and 500 μl of 5% CF_3_COOH in H_2_^18^O were heated at 120 °C for 40 hours in sealed tubes. Further, samples were purified using solid-phase extraction on Bond Elut PPL cartridges (Agilent Technologies) according to Zherebker *et al*.^54^. The sample treatment assured back-exchange of labile protons in case of H/D exchange. The final solutions were obtained *via* methanol elution. HDX of mobile protons was conducted by dilution of the methanol solution twice with D_2_O before further analysis.

All CHA and PHA samples were analyzed using a commercial 7 Tesla LTQ FT Ultra mass spectrometer (Thermo Electron Corp., Bremen, Germany) located at the Institute of Biochemical Physics of RAS (Moscow, Russia). Mass-spectra of native and labeled CHM samples were acquired on 7T FT MS Bruker Apex Ultra with harmonized cell (Bruker Daltonics) located at the Institute of Biomedical Chemistry (Moscow). All experiments were performed using negative electrospray ion mode. Analytical conditions for spectra acquisition and formulae calculation are described in details in our previous studies^39,54,57,58^. The data from the labeling experiments were processed following an algorithm that was described in our previous work^20^. It implies extraction of peaks related to exchange series of individual CHM constituents from the full mass spectrum. Those series are produced by peaks with m/z differences of 1.006277 and 2.004245, which correspond to the substitution of a proton with a deuteron and oxygen exchange, respectively.

### Biology

#### Cells and viruses

Porcine embryo kidney (PEK) cell line was maintained at 37 °C in medium 199 (FSBSI “Chumakov FSC R&D IBP RAS”, Russia) supplemented with 5% fetal bovine serum (FBS, Invitrogen). RD (rhabdomiosarcoma) cell line originated from NIBSC (UK) was maintained at 37 °C in EMEM with doubled amino acids and vitamins (2× EMEM, FSBSI “Chumakov FSC R&D IBP RAS”, Russia) supplemented with 5% FBS (Invitrogen) and penicillin (100 U/mL). Tick-borne encephalitis virus strain Absetarrov (GenBank access no. KU885457.1) was from the laboratory collection of FSBSI “Chumakov FSC R&D IBP RAS”. Reference vaccine strain Sabin 1 of poliovirus type 1 (GenBank access no. V01150) is from Moscow RRL Polio collection originated from NIBSC (UK). Enterovirus A71, isolate 46973 (GenBank accession no. KJ645808), was isolated from a patient with acute flaccid paralysis in 2013 in Russia. Echovirus 30, isolate 48461 (GenBank accession no. MK704489), was isolated from a patient with enteroviral meningitis in 2013 in Russia.

#### Cell toxicity assay

PEK cells toxicity assay: The protocol for cytotoxicity test in PEK cells was adopted from ref.^33^. PEK cells were seeded and incubated for 72 h at 37 °C. Two-fold dilutions of the HS sample stocks (concentration 1 g/L) were prepared in medium 199 in Earle solution to obtain final concentrations starting from 10 μg/mL. Equal volumes of HS sample dilutions were added to the cells in four replicates. Control cells were treated with the same sequential concentrations of KOH as in HS sample dilutions, in four replicates. After incubation at 37 °C on days 1 or 7, CC_50_ values were calculated according to the Karber method^59^.

RD cells toxicity assay: Eight 2-fold dilutions of stock solutions of the HS samples (concentration of 1 g/L) were prepared in 2×EMEM medium to obtain a final concentration series starting from 20.8 μg/mL. For cell control, the same sequential concentrations of KOH (0.03 M) were mixed with the equal volume of the culture medium. Afterwards, the RD cell suspension in 2×EMEM containing 5% FBS was added. Cells were incubated at 36.5 °C for 7 days. Cell morphology and vitality were assessed on days 1 (CC_50_ (24 h)) or 7 (CC_50_ (7 d)) visually, cytopathic changes were registered. CC_50_ was calculated according to the Karber method^59^.

#### Activity assays

TBEV plaque reduction test: Anti-TBEV activity test was performed as described previously^10^. Four-fold dilutions of the HS samples were preincubated with the virus (20–40 PFU) (EC_50_pre_) or added to the PEK cells monolayer simultaneously with the virus (EC_50_sim_) in 24-well plates. The same sequential concentrations of KOH and previously investigated compound 3-amino-7,7-dimethyl-2-(4-methylbenzoyl)-5H,6H,7H,8H-selenopheno[2,3-*b*]quinolin-5-one^5^ were used as a negative and positive controls, respectively. The plates were incubated for 1 h and overlaid with 1.26% methylcellulose. After 6 days, cells were fixed with ethanol and stained with 0.4% gentian violet. EC_50_ values were calculated according to the Reed and Muench method^60^.

EV cytopathic effect inhibition test: Cytopathic effect inhibition test against representatives of *Enterovirus* genus was performed as described previously^12^. Eight 2-fold dilutions of stock solutions of the HS samples (concentration of 1 µg/mL) were prepared in 2×EMEM medium to obtain a final concentration series starting from 50 μg/mL. The dilutions were mixed with equal volumes of the enterovirus suspension containing 100 TCID_50_ (50% tissue culture infective dose) in four replicates. After 1 hour incubation at 36 °C the RD cell suspension in 2×EMEM medium containing 5% FBS was added to the experimental mixtures. After a 5-day incubation at 37 °C, cytopathic effect (CPE) was visually assessed. The virus titre was calculated according to the Karber method^59^.

#### Chemoinformatics

Data processing was carried out using Python 2.7, NumPy 1.14.3, Seaborn 0.8.1, Pandas 0.23.0, MatPlotLib 2.2.2. Database management was carried out either in InstantJChem 17.2.6.0^61^ or DataWarrior 4.7.2^62^. MySQL version of ChEMBL 20 was accessed through MySQL Workbench (v. 6.3) interface. FTICR MS data were visualized using heatmaps and van Krevelen diagrams (relationship of H/C versus O/C atomic ratios)^40^. For the functional group analysis, the fully automated algorithm suggested in ref.^63^ was used. We used the implementation of this algorithm available in RDKit v. 2018.03.4.

HS formula space enumeration: The virtual HS-like formula space was generated using a Python 2.7 script implementing coin change problem algorithm. The source code is available in GitHub repository: https://github.com/AxelRolov/HS_formulae_generation. Given the prevalence of C, H, O atoms in elemental composition of natural HS^39^ and for simplifying calculations, only C, H, O atoms were used for formula generation. Elements were represented by their nominal atomic masses (12, 1, 16). All possible C_x_H_y_O_z_ formulae were generated in the range of molecular weights from 200 to 800 g/mol, consistent with the typical analytical window of FTICR MS for HS^40^. A total of 231,546 formulae was generated and subsequently filtered using numeric elemental constraints for typical HS-like formula space: 0.27 ≤ H/C ≤ 2.2, 0 < O/C ≤ 1^64^. There were 22,618 formulae left. To check the possibility of existence of molecular graphs corresponding to generated formulae we applied Senior’s rules^41–43^. Among generated formulae 1,001 did not pass Senior’s rules filter. Thus, the final number of formulae was 21,617 (Supplementary File S5).

The assigned formulae for HS samples were plotted into Van Krevelen diagrams which represent relationship of H/C ratio versus O/C ratio^40^. We used the obtained diagrams for generating numerical descriptors of the chemical space occupied by the isolated humic fractions. For this purpose, we applied cell-based partitioning approach and discretized the Van Krevelen diagram into 20 rectangular cells^46^. The cell-based distribution of experimental points was calculated by quantifying intensity-weighted population density of each cell (*D*_*k*_) as expressed by Eq. 1 below:

1 $${D}_{k}=\frac{\mathop{\sum }\limits_{i=1}^{{N}_{k}}{I}_{i}}{\mathop{\sum }\limits_{j=1}^{N}{I}_{j}},k=1,2,\ldots 20$$

where *D*_*k*_ is the intensity weighted population density of the cell *k*; *N* is the total number of points in the Van Krevelen diagram; *N*_*k*_ is the number of points belonging to the cell *k*; *I*_*j*_ is the intensity of point *j*; *I*_*i*_ – intensity of the point *i* belonging to the cell *k*. The obtained densities for two pairs of coal samples CHA-CHM were used for Spearman’s rank correlation coefficient calculation.

Fingerprint generation and heatmap-based visualization: For similarity analysis of the HS samples, the Boolean fingerprints were generated for each sample by matching the generated HS-like formulae with the FTICR MS formula assignments for each sample (Supplementary File S6). The fingerprint was constructed by setting 1 if the formula was detected in the sample by FTICR MS, and by setting 0 if the formula was not found in the FTICR MS assignments. For the heatmap visualization, the fingerprints were cut to the length of 6502 (number of different formulae in all the samples). One thousand fingerprints containing randomly positioned 2347 (mean number of formulae in natural HS samples) ‘1’ values across 6502 positions (‘Random’ fingerprints) were generated. Tanimoto indices of all samples’ fingerprints against all Random fingerprints were calculated.

ChEMBL data mining: MySQL edition of ChEMBL 20 was used for data mining. ChEMBL dump file was put into a local MySQL database. Molecular formulae (*full_molformula* field) and primary keys (*molregno* field) were extracted from ChEMBL *compound_properties* table with an SQL query. The generated HS-like formulae containing C, H, O atoms were searched in ChEMBL using a Python script (Supplementary File S7). There were 6189 entries of *full_molformula* field corresponding to 71,380 distinct *molregno* entries. Among others, ChEMBL formulae were extracted, containing non-covalently bound fragments (delimited by ‘.’ sign), of which at least one fragment formula matched the formula query. Such formulae (89 *full_molformula* entries and 98 *molregno* entries) were manually analysed. Formulae containing Re, Ru or Co atoms bound with organic counterpart were deleted as non-relevant (there were no structures nor relevant virus-related bioactivities available in ChEMBL). Formulae containing two atoms of Na or K (11 *full_molformula/molregno* entries) were also stripped as the information related to such formulae was retrieved on the next stage. There were 71,325 *molregno* entries left.

As we used only C_x_H_y_O_z_ formulae for the search, the potentially useful data on the activity of the salts of organic acids could be missed. To include this information, we used the data from ChEMBL table *molecule_hierarchy*. There are two fields in this table: *molregno*, containing the foreign key for *compounds* table, and *parent_molregno*, containing parent compound of *molregno*, generated by the standardisation procedure. We searched both these fields for *molregno* entries extracted on the previous stage. Again, all formulae containing non-covalently bound fragments were manually analyzed. Formulae containing organic molecule with the metal counterpart were preserved and converted to C_x_H_y_O_z_ form, as well as adducts of organic fragment with NH_3_, while all other formulae containing non–CHO elements were stripped. All the extracted *parent_molregno/molregno* identifiers were then concatenated in one list (71,864 *molregno* entries, Supplementary File S8). The antiviral activity data and compound structures were extracted from ChEMBL or ViralChEMBL via an SQL query using *molregno* as a key.

Scaffold analysis: For the scaffold analysis the structures extracted from ChEMBL were divided into two groups: structures related to formulae within FTICR MS assignments for HS samples and structures related to generated formulae. Murcko scaffolds were generated in DataWarrior 4.7.2.

## Supplementary information

Supplementary Files

## Data Availability

FTICR MS datasets analysed during the current study are available from the corresponding authors on reasonable request. All other data generated or analysed during this study are included in this published article (and its Supplementary Information Files).
