# Supplementary material for: Examination of molecular space and feasible structures of bioactive components of humic substances by FTICR MS data mining in ChEMBL database
Source: Sci Rep. 2019 Aug 19;9:12066. doi: 10.1038/s41598-019-48000-y (PMC6700089; doi:10.1038/s41598-019-48000-y)
Supplement: Supplementary file 1 — Supplementary Files [file 41598_2019_48000_MOESM1_ESM.zip › SupplementaryFiles/Supplementary File S1.pdf]

Supplementary Materials

Examination of molecular space and feasible structures of bioactive components of humic substances by FTICR MS data mining in ChEMBL database

Alexey A. Orlov[a,b,c], Alexander Zherebker[b,c], Anastasia A. Eletskaya[a,d], Viktor S. Chernikov[a], Liubov I. Kozlovskaya[a,e], Yury V. Zhernov[f], Yury Kostyukevich[b], Vladimir A. Palyulin[c], Eugene N. Nikolaev[b], Dmitry I. Osolodkin[a,c,e]*, Irina V. Perminova[c]*

[a] FSBSI "Chumakov FSC R&D IBP RAS", Moscow 108819, Russia, e-mail: dmitry_o@qsar.chem.msu.ru

[b] Skolkovo Institute of Science and Technology, Moscow, 143026, Russia

[c] Department of Chemistry, Lomonosov Moscow State University, Moscow 119991, Russia, e-mail: iperm@med.chem.msu.ru

[d] Department of Fundamental Medicine, Lomonosov Moscow State University, Moscow 119991, Russia

[e] Sechenov First Moscow State Medical University, Moscow 119991, Russia

[f] State Research Center "Institute of Immunology" of the Federal Medical-Biological Agency of Russia, Moscow 115478, Russia

Keywords: data mining, antiviral activity, humic substance, flaviviruses, ChEMBL, FTICR MS, structural fragments, isotopic exchange

Table S1. Elemental composition of HS samples studied.

Sample	Element content, %				TBEV EC_{50_pre} (µg/ml)
	C	H	N	O	
CHA-SH4	49.07	3.96	1.83	45.14	0.74 ± 0.15
CHA-Pow	57.06	4.04	0.92	37.98	0.26 ± 0.14
CHA-Gl	56.99	3.44	2.94	36.63	0.30 ± 0.19
CHM-Irk	55.73	3.58	1.50	39.19	0.14 ± 0.08
CHM-Gl	42.76	2.92	3.34	50.98	0.514 ± 0.025
CHM-Pow	50.07	2.17	1.01	46.75	0.808 ± 0.016
PHA-TTL	50.09	4.37	2.06	43.48	0.7 ± 0.3
PHA-T7	52.13	4.68	1.32	41.87	0.9 ± 0.1
HQ-FA	51.46	4.10	0.00	44.44	>10
MHQ-FA	52.67	4.26	0.00	43.07	>10

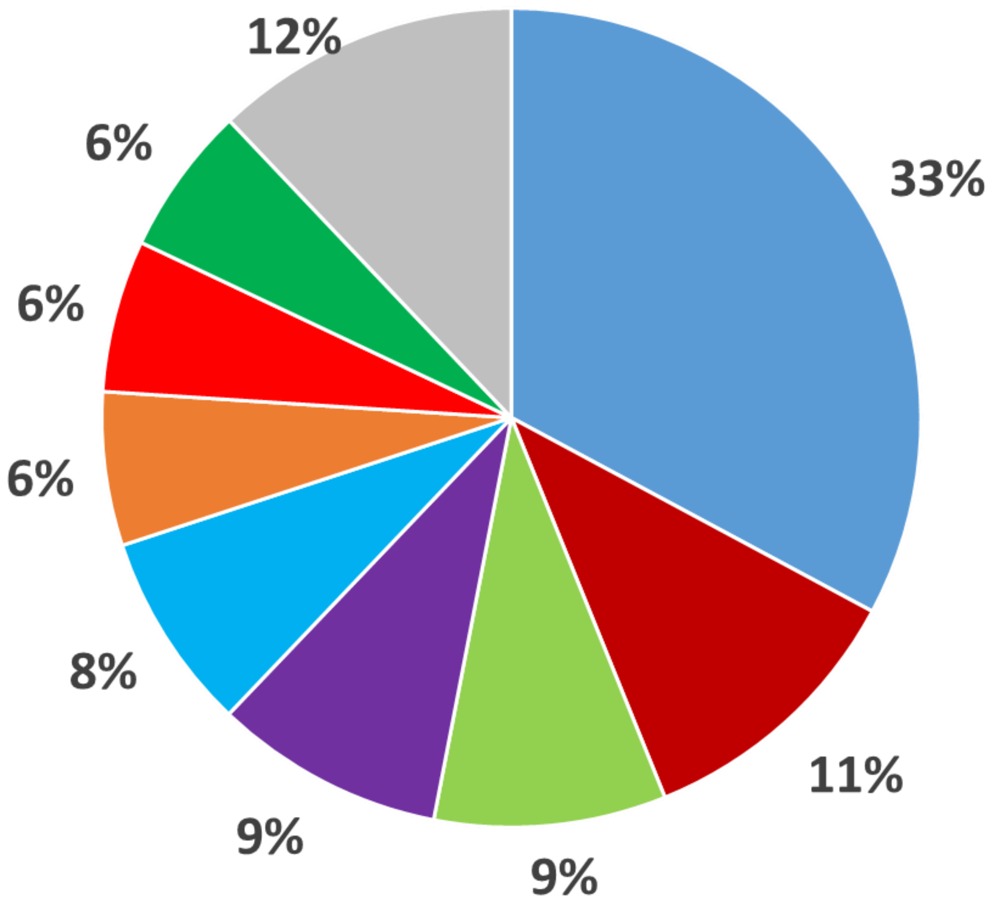

Supplementary Figure S1. The percentage of ChEMBL antiviral activity data points for the HS-like subset corresponding to the viruses belonging to the families listed below: Retroviridae (blue), Flaviviridae(dark red), Orthomyxoviridae (light green), Filoviridae (violet), Herpesviridae (cyan), Poxviridae (orange), Arenaviridae (red), Hepadnaviridae (dark green), other (grey).

	D1	D2	D3	D4	D5	D6	D7	D8	D9	D10	D11	D12	D13	D14	D15	D16	D17	D18	D19	D20	EC50
D1	1	1	0.8	0.4	0.6	0.8		-0.4	-0.4	1	-0.8	-0.8	-0.6	-0.26	0.26	-0.8	-0.4	0.95	0.95	?	-0.6
D2	1	1	0.8	0.4	0.6	0.8		-0.4	-0.4	1	-0.8	-0.8	-0.6	-0.26	0.26	-0.8	-0.4	0.95	0.95	?	-0.6
D3	0.8	0.8	1	0.8	0.8	0.6	-0.4			0.8	-1	-1	-0.8	-0.26	0.77	-1	-0.8	0.74	0.74	?	-0.8
D4	0.4	0.4	0.8	1	0.4		-0.2	0.6	0.6	0.4	-0.8	-0.8	-0.4	0.26	0.77	-0.8	-1	0.21	0.21	?	-0.4
D5	0.6	0.6	0.8	0.4	1	0.8	-0.8	-0.4	-0.4	0.6	-0.8	-0.8	-1	-0.77	0.77	-0.8	-0.4	0.74	0.74	?	-1
D6	0.8	0.8	0.6		0.8	1	-0.4	-0.8	-0.8	0.8	-0.6	-0.6	-0.8	-0.77	0.26	-0.6		0.95	0.95	?	-0.8
D7			-0.4	-0.2	-0.8	-0.4	1	0.2	0.2		0.4	0.4	0.8	0.77	-0.77	0.4	0.2	-0.21	-0.21	?	0.8
D8	-0.4	-0.4		0.6	-0.4	-0.8	0.2	1	1	-0.4			0.4	0.77	0.26		-0.6	-0.63	-0.63	?	0.4
D9	-0.4	-0.4		0.6	-0.4	-0.8	0.2	1	1	-0.4			0.4	0.77	0.26		-0.6	-0.63	-0.63	?	0.4
D10	1	1	0.8	0.4	0.6	0.8		-0.4	-0.4	1	-0.8	-0.8	-0.6	-0.26	0.26	-0.8	-0.4	0.95	0.95	?	-0.6
D11	-0.8	-0.8	-1	-0.8	-0.8	-0.6	0.4			-0.8	1	1	0.8	0.26	-0.77	1	0.8	-0.74	-0.74	?	0.8
D12	-0.8	-0.8	-1	-0.8	-0.8	-0.6	0.4			-0.8	1	1	0.8	0.26	-0.77	1	0.8	-0.74	-0.74	?	0.8
D13	-0.6	-0.6	-0.8	-0.4	-1	-0.8	0.8	0.4	0.4	-0.6	0.8	0.8	1	0.77	-0.77	0.8	0.4	-0.74	-0.74	?	1
D14	-0.26	-0.26	-0.26	0.26	-0.77	-0.77	0.77	0.77	0.77	-0.26	0.26	0.26	0.77	1	-0.33	0.26	-0.26	-0.54	-0.54	?	0.77
D15	0.26	0.26	0.77	0.77	0.77	0.26	-0.77	0.26	0.26	0.26	-0.77	-0.77	-0.77	-0.33	1	-0.77	-0.77	0.27	0.27	?	-0.77
D16	-0.8	-0.8	-1	-0.8	-0.8	-0.6	0.4			-0.8	1	1	0.8	0.26	-0.77	1	0.8	-0.74	-0.74	?	0.8
D17	-0.4	-0.4	-0.8	-1	-0.4		0.2	-0.6	-0.6	-0.4	0.8	0.8	0.4	-0.26	-0.77	0.8	1	-0.21	-0.21	?	0.4
D18	0.95	0.95	0.74	0.21	0.74	0.95	-0.21	-0.63	-0.63	0.95	-0.74	-0.74	-0.74	-0.54	0.27	-0.74	-0.21	1	1	?	-0.74
D19	0.95	0.95	0.74	0.21	0.74	0.95	-0.21	-0.63	-0.63	0.95	-0.74	-0.74	-0.74	-0.54	0.27	-0.74	-0.21	1	1	?	-0.74
D20	?	?	?	?	?	?	?	?	?	?	?	?	?	?	?	?	?	?	?	1	?
EC50	-0.6	-0.6	-0.8	-0.4	-1	-0.8	0.8	0.4	0.4	-0.6	0.8	0.8	1	0.77	-0.77	0.8	0.4	-0.74	-0.74	?	1

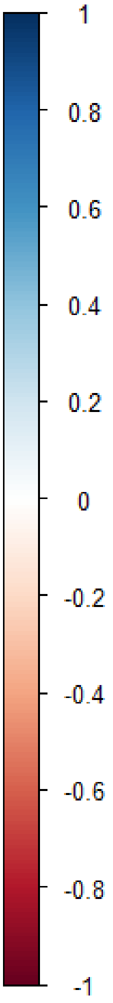

Supplementary Figure S2. Spearman correlation matrix of cell’s population density in van Krevelen diagram and EC_{50} values.

Table S2. Lengths of isotopic exchange series for selected formulae from CHM-POW sample determined by FTICR MS.

M, Da	m/z	relativeIntensity	DBE	Formula	C	H	O	18O/16O	D2O	H-labile	DCl	DBE
326.043	325.036	0.426023375	13	C17H10O7	17	10	7	6	4	5	5	14
426.204	425.197	0.232123983	11	C25H30O6	25	30	6	4	3	4	5	12
440.22	439.213	0.178374948	11	C26H32O6	26	32	6	4	2	3	5	12
424.189	423.182	0.176617729	12	C25H28O6	25	28	6	5	3	4	4	13
452.22	451.213	0.172874309	12	C27H32O6	27	32	6	5	3	4	5	13
438.204	437.197	0.165943974	12	C26H30O6	26	30	6	6	3	4	5	13
498.262	497.255	0.161816483	11	C29H38O7	29	38	7	5	3	4	3	12
422.173	421.166	0.154447442	13	C25H26O6	25	26	6	5	2	3	3	14
436.189	435.182	0.140224781	13	C26H28O6	26	28	6	5	3	4	4	14
466.235	465.228	0.137434612	12	C28H34O6	28	34	6	4	3	4	3	13
434.173	433.166	0.127546662	14	C26H26O6	26	26	6	4	3	4	4	15
310.048	309.041	0.122811346	13	C17H10O6	17	10	6	5	2	3	3	14
396.194	395.187	0.112899191	11	C24H28O5	24	28	5	4	2	3	5	12
480.251	479.244	0.112067402	12	C29H36O6	29	36	6	4	3	4	4	13
284.032	283.025	0.110586854	12	C15H8O6	15	8	6	5	3	4	2	13
410.209	409.202	0.108807947	11	C25H30O5	25	30	5	4	2	3	5	12
382.178	381.171	0.108787601	11	C23H26O5	23	26	5	4	2	3	5	12
424.225	423.218	0.100762883	11	C26H32O5	26	32	5	4	2	3	4	12
366.147	365.14	0.098196634	12	C22H22O5	22	22	5	4	4	5	4	13
368.162	367.155	0.094269008	11	C22H24O5	22	24	5	4	2	3	5	12
354.147	353.14	0.086461864	11	C21H22O5	21	22	5	4	4	5	4	12
452.256	451.249	0.084495256	11	C28H36O5	28	36	5	3	2	3	3	12
392.162	391.155	0.0789206	13	C24H24O5	24	24	5	3	3	4	3	14
306.053	305.046	0.076787796	14	C18H10O5	18	10	5	4	2	3	2	15
366.183	365.176	0.033010102	11	C23H26O4	23	26	4	3	2	3	3	12
